# Rebuttal to the letter “Assessment of COVID-19-related right ventricular morphological and functional alterations and evaluation of their impact on the course of the disease”

**DOI:** 10.1186/s13613-024-01292-4

**Published:** 2024-04-24

**Authors:** Mathieu Jozwiak

**Affiliations:** 1 Service de Médecine Intensive Réanimation, CHU de Nice, 151 route Saint Antoine de Ginestière, Nice, 06200 France; 2 UR2CA - Unité de Recherche Clinique Côte d’Azur, Université Côte d’Azur, Nice, France

I would like to thank Dr DANDEL for his interest in our article and for his letter, in which he raises three important points.

Firstly, I fully agree that right ventricular (RV) assessment is of utmost importance in patients with COVID-19, in whom RV injury is frequent, because the mismatch between RV systolic function and increased pulmonary vascular resistance due to small vessel thrombosis and pulmonary embolism rather than to hypoxic vasoconstriction, which is known to be lower or even absent, is associated with mortality. Thus, repeated echocardiographic examinations during intensive care unit (ICU) stay are needed for early identification of patients at risk for COVID-19-associated RV injury [1].

Secondly, the lack of specific assessment of the severity of tricuspid regurgitation, which we acknowledged as a limitation of our study, was mentioned as a confounding factor for the assessment of RV systolic function. It was therefore suggested that the similar values of tricuspid annular plane systolic excursion (TAPSE) and RV fractional area change (RV FAC) that we found on ICU admission between patients with isolated RV dilation and patients without RV injury could potentially be explained by a difference in severity of tricuspid regurgitation between the two groups of patients. Although this explanation cannot be strictly excluded, there are other potential explanations for these unexpected findings at first glance. Physiologically, there is probably a severity gradient between isolated RV dilatation and acute cor pulmonale, with isolated RV dilation initially thought to be a functional adaptative mechanism to maintain cardiac output despite increased RV afterload, according to Frank-Starling’s law. At this stage, the RV systolic function is still preserved, which explains the similar values in TAPSE and RV FAC that we found between patients with isolated RV dilation and patients without RV injury. Impairment of RV systolic function would therefore only appear in a second time when RV afterload is persistently increased and would be associated with more marked RV dilation. This hypothesis is consistent with the fact that in our study, patients with RV dilation and RV systolic dysfunction on ICU admission had a higher RV/left ventricular (LV) end-diastolic areas ratio than patients with isolated RV dysfunction. Furthermore, LV systolic function is part of RV systolic function, as both ventricles are morphologically and functionally inextricably linked [2]. In this regard, it is noteworthy that patients with isolated RV dilation on ICU admission tended to have a higher LV ejection fraction than patients without RV injury.

Thirdly, it was highlighted that patients with isolated RV dilation on ICU admission had a higher mortality rate than patients with RV systolic dysfunction regardless of RV dilation. This result is consistent with the existing literature, which has shown that early isolated RV dilation is associated with in-hospital mortality in patients with COVID-19 [3]. As suggested by Dr DANDEL, this early isolated RV dilation could illustrate a lower RV adaptability to increased RV afterload, thus explaining this poorer prognosis. This physiological hypothesis is consistent with our findings, which showed in patients with isolated RV dilation on ICU admission a trend towards RV-pulmonary arterial uncoupling, evidenced by a decrease in the TAPSE/systolic pulmonary artery pressure ratio, a reliable echocardiographic surrogate of the reference standard of RV–pulmonary arterial coupling [4]. Therefore, early isolated RV dilation in patients with COVID-19 should be considered a red flag, as it could be an early marker of a higher risk of mortality, even though in our study only the combination of RV dysfunction with RV dilation or acute cor pulmonale, but not isolated RV dilation, was independently associated with Day-28 mortality. However, this result may have other explanations than this physiological one. Given the limited sample size in each patient group, this higher mortality rate in patients with isolated RV dilation on ICU admission should be interpreted with caution. Furthermore, unlike previous studies, we combined all three echocardiographic parameters of RV systolic function for the diagnosis of RV dysfunction, as measurement of RV longitudinal function alone by TAPSE failed to detect RV impairment in patients with COVID-19 [5]. If TAPSE only had been considered, a significant proportion of patients considered to have RV systolic dysfunction on ICU admission would have been considered not to have RV injury, which could explain the lower mortality rate found in these patients. Finally, as 10% of patients in our cohort experienced different RV injury patterns, the RV injury pattern experienced on ICU admission was not necessarily the most severe RV injury pattern during ICU stay, whereas only this most severe pattern was considered for the multivariate analysis of mortality.

To conclude, particular attention should be paid to RV function in critically ill patients with COVID-19 with early and repeated echocardiographic examinations, as RV injury is common and the most severe RV injury patterns are associated with mortality.

## Data Availability

Not applicable.

## Abbreviations

ICU: intensive care unit
LV: left ventricular
RV: right ventricular
TAPSE: tricuspid annular plane systolic excursion
RV FAC: RV fractional area change
